# Does ACPA-negative RA consist of subgroups related to sustained DMARD-free remission and serological markers at disease presentation? Comment on article by Boeters DM et al.

**DOI:** 10.1186/s13075-020-2106-5

**Published:** 2020-01-31

**Authors:** Alfonse T. Masi, Roy Fleischmann

**Affiliations:** 1 Department of Medicine, University of Illinois College of Medicine at Peoria, One Illini Drive, Peoria, IL USA; 2 Co-Medical Director Metroplex Clinical Research Center, University of Texas Southwestern Medical Center, 8144 Walnut Hill Lane Suite 810, Dallas, TX 75231 USA

**Keywords:** Rheumatoid arthritis, Anti-citrullinated protein antibody (ACPA), Multi-biomarker disease activity (MBDA) score, Remission

Letter to the Editor

We read with interest the article by Boeters DM et al. [1]. We would like to clarify the significant findings and suggest further research needed to validate the novel conclusion. In ACPA-negative patients, 1 sustained DMARD-free remission (SDFR) occurred over 5 years follow-up in 17 (6%) with baseline low (< 30) MBDA score vs approximately 50% remissions in both moderate (30–44) and high (> 44) MBDA score patients ([1], Fig. 1). All ACPA-*positive* RA patients had low percentages of SDFR and *no* difference was found by baseline MBDA score category [1]. Percentages of 3 MBDA categories did *not* differ (*p* = 0.470) between the ACPA-positive and ACPA-negative groups [1].

SDFR was recently reported by ACPA-negative vs ACPA-positive patients in the *total* Leiden early arthritis cohort (1993–2016; *n* = 1296) [2], from which the Boeters et al. study [1] was the most recent inclusion subgroup (2011–2016). In the total inclusion period (1993–2011), SDFR occurred between 5 and 15% in ACPA-positive RA vs 40 to 50% in the ACPA-negative RA [2], as in Boeters et al. [1].

Unexpectedly, in multivariate analyses ([1], Table 2), the 95 ACPA-negative RA patients with high (> 44) baseline scores had *greater* DMARD-free remission than the 17 reference patients with low (< 30) MBDA scores (*p* = 0.041). If MBDA were truly a marker of disease activity, one might expect low rather than high MBDA to predict SDFR. Alternatively, if ACPA-negative RA does consist of subgroups [1], its documentation will require further serological study in separate cohorts [2, 3] or search for genetic markers [3]. Confounding variables should be excluded, possibly clinical features related to age at onset, which was found to be a significant (*p* = 0.036) predictor of SDFR ([1], Table 2) and other disease variables not studied. Is it conceivable that this anomaly [1] is due to chance occurrence in a small sample size study leading to an incorrect conclusion, especially when borderline (*p* = 0.041) statistical correlation is found [1]?

A critical review of the value of multibiomarker disease activity score to predict remission in RA was recently published [4]. The challenging question is whether or not baseline MBDA (or serological markers) are being overinterpreted or overstated with respect to outcomes (or disease subgroups) was critically analyzed [4].

## Data Availability

Data are in published article and reference citations.

## References

[1] Boeters DM, Burgers LE, Sasso EH, Huizinga TWJ, van der Helm-van Mil AHM (2019). ACPA-negative RA consists of subgroups: patients with high likelihood of achieving sustained DMARD-free remission can be identified by serological markers at disease presentation. Arthritis Res Ther.

[2] Matthijssen XM, Niemantsverdriet E, Huizinga TW, van der Helm-van Mil AHM. ACPA-positive patients benefited more than ACPA-negative patients; 25 year results of a longitudinal cohort study. Presentation 2871 at the 2019 American College of Rheumatology Meeting, Atlanta GA (pps 5098–5100 in <https://acrabstracts.org/wp-content/uploads/2019/10/2019-Abstract-Supplement.pdf>).

[3] Hedström AK, Rönnelid J, Klareskog L, Alfredsson L (2019). Complex relationships of smoking, HLA-DRB1 genes, and serologic profiles in patients with early rheumatoid arthritis: update from a Swedish population-based case-control study. Arthritis Rheumatol.

[4] Fleischmann R (2019). Value of the multibiomarker of disease activity score to predict remission in RA: what does the evidence show?. J Rheumatol.

